# Exploring the Therapeutic Potential, Ethnomedicinal Values, and Phytochemistry of *Helianthus tuberosus* L.: A Review

**DOI:** 10.3390/ph17121672

**Published:** 2024-12-11

**Authors:** Ruvimbo Faith Tapera, Xavier Siwe-Noundou, Leshweni Jeremia Shai, Shoeshoe Mokhele

**Affiliations:** 1 Department of Pharmaceutical Sciences, School of Pharmacy, Sefako Makgatho Health Sciences University, Pretoria 0204, South Africa; 201906818@swave.smu.ac.za (R.F.T.); xavier.siwenoundou@smu.ac.za (X.S.-N.); 2 Department of Biomedical Sciences, Faculty of Science, Tshwane University of Technology, Arcadia Campus, Pretoria 0183, South Africa; shailj@tut.ac.za

**Keywords:** *Helianthus tuberosus* L., Jerusalem artichoke, inulin, phytochemicals, pharmacological properties

## Abstract

*Helianthus tuberosus* L. (Jerusalem artichoke) tubers and aerial parts possess both nutritional and therapeutic properties. The Jerusalem artichoke has been utilized for various applications, including its use as a functional food source, a reservoir of bioactive compounds, and a raw material to produce biofuels. Moreover, the Jerusalem artichoke is a rich source of an indigestible polysaccharide called inulin, which serves as a prebiotic that improves gastrointestinal health. This plant has been used globally throughout history as a dietary supplement, for pain treatment, to reduce swelling, and to boost the immune system, as well as to treat skin wounds in folk medicine. It is an abundant source of bioactive compounds, such as phenolic acids, coumarins, and flavonoids, which are known to exert pharmacological activities, including antioxidant, antimicrobial, and anti-inflammatory properties. The literature on its potential as an antidiabetic, anticancer, anti-fungistatic, antiviral, and anti-obesity agent, among others, is scanty. This review aims to provide a comprehensive overview of *Helianthus tuberosus* L.’s traditional uses, nutritional properties, secondary bioactive compounds, and pharmacological properties to further explore its health benefits.

## 1. Introduction

Society has been utilizing herbal medicines to control, manage, and treat many medical conditions over the years [1]. For thousands of years, medicinal plants have been used by communities worldwide as a healing resource, and they continue to be a key component of present-day healthcare for around 85% of the global population [2]. Medicinal plants play a significant role in drug discovery, as evidenced by the observation that approximately 80% of synthetic drugs were derived or inspired by plant-derived natural compounds [3]. Therefore, medicinal plants have a diverse range of phytochemicals which may be effective in developing new therapeutic compounds against a spectrum of human diseases [4,5,6].

*Helianthus tuberosus* L. is among the medicinal plants that are currently considered worldwide. *Helianthus tuberosus* L. is a perennial species native to North America. This plant has since been introduced to Europe and Asia as a cultivated crop and is now grown in these regions as well as in other parts of the world [7,8]. Globally, *H. tuberosus* L. has approximately 2.5 million hectares devoted to its cultivation. The plant is mainly cultivated in the United States of America, where it utilizes 700,000 hectares; in France, where it utilizes 250,000 hectares with a yield of 7.5 million tons; and lastly in Austria, where it utilizes 130,000 hectares [9]. It is also cultivated in Italy, Spain, and Germany as a vegetable crop [10].

As the plant was cultivated by native Americans first, they referred to the plant as sun roots [11]. When the plant was introduced to other parts of the world, diverse common names were ascribed in various languages. The current, most utilized names are included in Table 1. *Helianthus tuberosus* L. also has a wide range of scientific synonyms, which reflect its taxonomic reclassification and its widespread cultivation and recognition. Some of these synonyms are also displayed in Table 1.

The genus *Helianthus* belongs to the family Asteraceae and the Asteroidean subfamily [20]. It consists of approximately fifty species, and only two species are cultivated because of their medicinal and nutritional values. These two economically significant species are *Helianthus tuberosus* L. and *Helianthus annus* [21]. *Helianthus annus* is an oilseed plant mainly cultivated for its nutritional value [22], while *Helianthus tuberosus* L. is a perennial tuber plant currently cultivated worldwide for its advantageous characteristics [23].

*Helianthus tuberosus* L. thrives in various soil types without the use of chemicals and/or fertilizers. It possesses beneficial characteristics, including resistance to frost and low maintenance requirements, and it is less susceptible to diseases and pests, which draws the attention of the researchers who utilize it [8,24,25].

**Figure 1:**
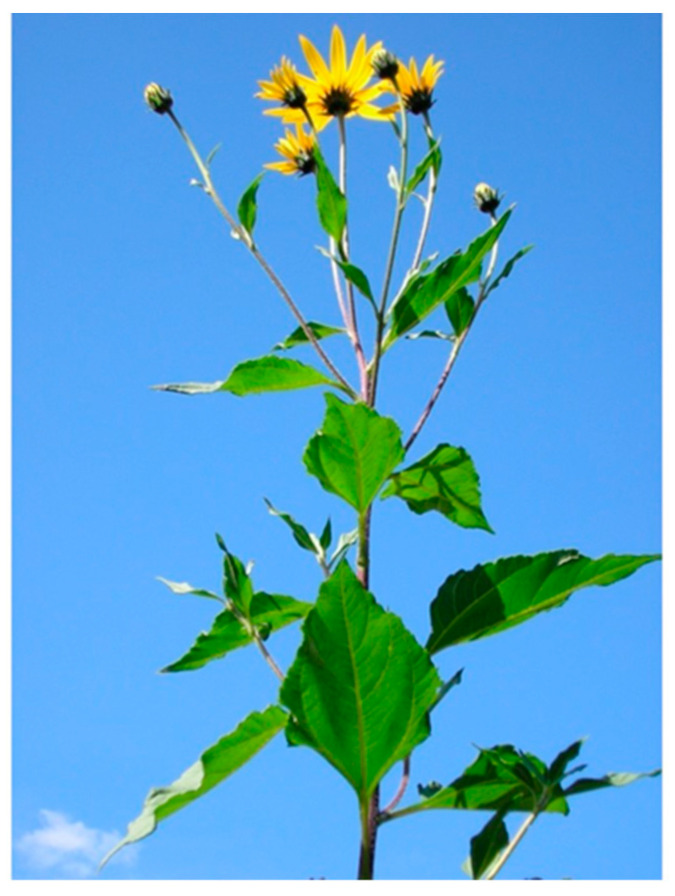
*Helianthus tuberosus* L., aerial part (flowers and leaves), image adapted from http://creativecommons.org/licenses/by-nc-nd/3.0/ accessed on 15 October 2024 [19].

The plant (Figure 1) grows well in semi-arid tropical areas [26]. It requires an annual precipitation of between 31 and 282 cm and an annual temperature range between 6.3 and 26.6 °C/43.3 to 79.9 °F (pH 4.5–8.2) [19]. The height of the plant is usually between 300 and 450 cm (stem), and the aerial parts of the plant include flowers, leaves, and stems [13,18].

**Figure 2:**
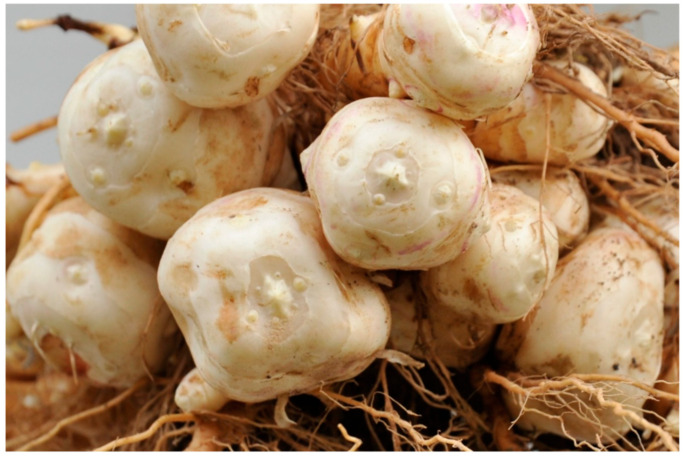
*Helianthus tuberosus* L, underground part (tubers, roots and stolon), image adapted from https://creativecommons.org/licenses/by/4.0/ accessed on 27 October 2024 [29].

The underground part of the plant comprises roots and stolon, as well as fleshy tubers (Figure 2) that can be of various shapes (round, pear-shaped, or oval) and sizes and bear a resemblance to those of potatoes [27]. According to Wang et al. [28], the color of the tubers varies between pale brown, dark brown, red, purple, and white depending on the climate and weather.

*Helianthus tuberosus* L., is one of the most underexplored medicinal plants with substantial therapeutic potential [30]. In folk medicine, *H. tuberosus* L. has been utilized for various medical applications, including immune system boosting, treatment of bone fractures, wound healing, the lowering of blood glucose levels in diabetic patients, the lowering of cholesterol and triglyceride levels in obese people, and the improvement of the digestive systems of individuals [31]. Overall, *H. tuberosus* L. exerts pharmacological properties, including antifungal, antioxidant, anti-inflammatory, anticarcinogenic, antimicrobial, antiviral, antidiabetic, anticancer, and body mass-reducing activities [32].

Moreover, the plant serves as a functional food ingredient, like inulin, fructose, and oligofructose, which are valued in the food industry for their roles in mitigating conditions such as type 2 diabetes mellitus and obesity [7,19]. Despite its benefits, significant gaps remain in the comprehensive understanding of its therapeutic potential, pharmacological mechanisms, and phytochemistry [33,34].

This review aims to bridge these gaps by analyzing and evaluating the existing literature on *H. tuberosus* L., with a focus on its phytochemistry, ethnomedicinal applications, nutritional composition, and pharmacological properties, and to provide a comprehensive resource detailing its integration into modern medicine.

## 2. Materials and Methods

This review mainly focuses on analyzing and reporting the scientific information published on the therapeutic effects, nutritional value, phytochemistry, and traditional uses of *H. tuberosus* L. The data were collected from reputable national and international journals and platforms, such as Science Direct, Google Scholar, Pub-Chem, NCBI-PubMed, ResearchGate, and Scopus, using search terms like “*Helianthus tuberosus* L.”, “Jerusalem artichoke”, “therapeutic properties”, and “phytochemistry”. The review encompasses the scientific literature published between 2014 and 2024. Ethical approval was unnecessary as previously published data were used.

## 3. Traditional Uses

*Helianthus tuberosus* L. has been traditionally used in various ways and applications. Firstly, the plant comprises edible tubers that can be consumed raw (crisp, sweet, crunchy texture when raw) or cooked (in salads and soups or stir-fried, roasted, boiled, or mashed like potatoes) [23]. It is a rich source of carbohydrates, protein, fiber, and other essential components that are beneficial for livestock and people [28]. The plant is also utilized as animal feed due to its nutritious content. It is usually used as a forage crop for animals [35]. The plant boosts the immune systems as well as the digestive systems of both animals and humans, through inulin which acts as a prebiotic.

*Helianthus tuberosus* L. is a raw material for a few functional foods; it can be used to target various functions of the human body beyond its basic nutritional effects. It is a rich source of inulin, fructose, and oligofructose [7]. Inulin is a low-calorie food that is usually white in appearance and usually has a sweet taste. It can be used to replace sugar and fat in dairy products as well as other baked goods to reduce calories [33]. This is beneficial in people with type 2 diabetes, obesity, and other related conditions. Fructose is a monomer of inulin and is known to have a low glycemic index (GI). Due to its low glycemic index, it is mainly used as a sweetener in place of glucose or sucrose in functional foods, which are also beneficial for people with obesity and type 2 diabetes [19,34].

Apart from its culinary uses, *H. tuberosus* L. is also used in folk medicine due to its potential health benefits owing to the presence of various essential components in the plant parts [36]. The tuber is used for pain treatment, skin wounds, swelling, promoting digestion, and the treatment of bone structures [28]. According to Sawicka et al. [9], other parts of the plant, such as the leaves and flowers, are used to treat joint diseases, arthritis, osteochondrosis, arthrosis, and bursitis. These parts are, however, underexplored. In certain cultures, the plant is used to treat ailments such as rheumatism, skin conditions (eczema and acne), and diabetes mellitus [37]. *Helianthus tuberosus* L. has also been traditionally used to treat constipation (laxative), to reduce fluid buildup in the body (diuretic) and/or increase secretion of bile from the liver (choleretic), and to improve detox capacity in the cells and tissues of the body [9].

Moreover, the inulin that is usually extracted from the plant is usually converted into fermentable sugars to produce biofuels such as ethanol and butanol [18,38], as well as to produce acids, including lactic acid, butyric acid, propionic acid, and citric acid [39]. Beyond these products, it can also be used to produce a range of value-added products in the biorefinery processes, including biodegradable polymers and biodegradable plastics. The bioenergy production by *H. tuberosus* L. offers a possible solution to the problem of reducing greenhouse gas emissions and the dependence on non-renewable sources of energy [40,41,42].

## 4. Market Value of *Helianthus tuberosus* L.

*Helianthus tuberosus* L. has gained significant attention in the global market due to its diverse applications in the nutraceutical, pharmaceutical, and functional food industries [43,44,45]. Known for its high inulin content, *H. tuberosus* L. has become a valuable source for producing prebiotics, natural sweeteners, and dietary supplements, particularly in the management of metabolic conditions such as diabetes and obesity, as well as for gut health [45].

Fructooligosaccharides (FOS) and inulin (which heavily relies on plants like *H. tuberosus* L.) are among the most prominent prebiotic components dominating the global market. In 2016, the market value for prebiotics was approximately USD 3.34 billion, with an estimated compound annual growth rate (CAGR) of 10.0% projected through 2025, signaling significant growth potential during the forecast period. The food and beverage industries are the largest consumers, accounting for 82% of the market share, while the animal feed sector also represents a significant segment, valued at USD 281.9 million in 2015. This reflects the growing demand and versatile applications of prebiotics across various industries [46]. This growth is driven by increasing consumer demand for plant-based and gut-friendly products. In addition, *H. tuberosus* L. is used in traditional medicine for its antioxidant, anti-inflammatory, and antimicrobial properties, further expanding its market potential in the herbal medicine sector [30].

Despite its market potential, the commercialization of *H. tuberosus* L. faces challenges, including the need for improved standardized processing methods and regulatory approvals [47]. However, a growing consumer preference for natural, sustainable products [23] is expected to boost its market value, making *H. tuberosus* L. a promising crop for the future of health and wellness industries.

## 5. Overview of the Chemical Composition and Bioactive Compounds

Reportedly, the tubers, leaves, and flowers of *Helianthus tuberosus* L. possess numerous essential components. Its chemical composition comprises carbohydrates, protein, fat, and a variety of other nutrient elements, such as potassium, vitamins, iron, and magnesium [48]. The plant is also a rich source of various bioactive compounds that account for its pharmacological and nutritional properties [49,50].

### 5.1. The Nutritional Value of Helianthus tuberosus L.

The extensive use of *H. tuberosus* L. as a food source for centuries is largely due to the nutritional value of its tubers [48]. Table 2 below shows the nutritional composition found in plant tubers, specifying the quantity and the method of quantification used.

### 5.2. Bioactive Secondary Metabolites

*Helianthus tuberosus* L. contains a variety of bioactive secondary metabolites, such as flavonoids (quercetin, kaempferol, and vanillin), phenolic acids (chlorogenic acid and ferulic acid) and sesquiterpenoids (angelylgrandifloric acid). These compounds exert several pharmaceutical properties, including antioxidant, anti-inflammatory, anticarcinogenic, and antimicrobial activities and many more [18,55,56]. However, the presence and quantity of these metabolites can differ depending on the geographical location of the plant, soil type, and climate conditions [57]. Additionally, the composition and existence of these metabolites can also be influenced by the type of solvents used during extraction [58]. Several studies have reported various compounds identified from *Helianthus tuberosus* L. plant extracts that were obtained through analytical procedures such as HPLC, GC-MS/MS, HPLC-UV, HPLC-NMR, and LC-MS/MS [59,60,61,62]. Table 3 shows examples of the previously reported compounds from different *Helianthus tuberosus* L. plant parts, their pharmacological properties, and their chemical structures.

## 6. Pharmacological and Therapeutic Properties of *Helianthus tuberosus* L. Plant

The presence of a variety of chemical compounds and inulin in *Helianthus tuberosus* L. is associated with important notable pharmacological and therapeutic qualities of the plant. The plant exhibits a variety of beneficial health properties that can help individuals with several medical conditions [39].

### 6.1. Anti-Obesity Properties

The anti-obesity properties are primarily associated with the rich content of inulin in *H. tuberosus* L. Inulin reduces obesity through several mechanisms, including metabolic regulation and appetite control. Inulin is a low-calorie prebiotic fiber responsible for delaying fat absorption, lowering triglyceride levels, and lowering blood glucose levels, as well as lowering cholesterol levels [30]. Inulin delays fat absorption, reduces triglyceride levels, and decreases cholesterol and blood glucose levels by inhibiting their synthesis and redistributing cholesterol from the plasma to the liver as well as by binding with bile acids and increasing their excretion [71]. It is a sweet-tasting soluble fiber, which makes it a good substitute for fat and sugar in recipes without contributing excess calories, thereby minimizing obesity [47]. The high fiber content in the plant aids in reducing food intake, which subsequently reduces weight and body mass index (BMI) in obese people [72]. Inulin from the plant also inhibits the overexpression of G-protein coupled receptor 43, thereby mitigating high-fat diet-induced obesity [73].

Coumarins (e.g., ayapin), phenolic acids (e.g., caffeic acid), and sesquiterpenes (e.g., heliangin) [37,58,70] exert anti-inflammatory effects, which help in reducing obesity-related inflammation. Antioxidants like rhamnazin, budlein A, and salicylic acid mitigate the oxidative stress associated with obesity by scavenging free radicals in the body [36,74].

A study investigated the effect of inulin from *H. tuberosus* L. on managing weight in forty-four ectopic fat subjects over a period of 18 weeks. The inulin group showed greater weight loss between weeks 9 and 18 (−2.3  ±  0.5% vs. −0.6  ±  0.4%, *p* = 0.012) and reduced hepatic (*p* = 0.02) and soleus muscle fat content (*p* < 0.05). Fasting glucose decrease was observed on week 9 (*p* = 0.005), while insulin concentrations remained unchanged [75]. In another study using obese C57BL/6J mice, polyherbal formulations containing *H. tuberosus* L. root powder reduced insulin resistance, fat deposition, and body weight while improving lipid profiles [76]. Moreover, *H. tuberosus* L. consumption before meals significantly lowered total and active glucose-dependent insulinotropic polypeptide (GIP) levels post-meal (*p* < 0.001) without affecting glucagon-like peptide-1 (GLP-1) [77].

Overall, *H. tuberosus* L. improves weight management through various mechanisms, including appetite control, regulation of metabolism, and reduction in inflammation [78], but the efficacy of these methods has not yet been confirmed.

### 6.2. Antidiabetic Properties

*Helianthus tuberosus* L. can exert antidiabetic properties through several mechanisms. *Helianthus tuberosus* L. exhibits antidiabetic properties, which are primarily due to the presence of soluble fibers in the plant, mainly in the form of inulin. These soluble fibers help in increasing the viscosity of nutrients in the small intestines, which reduces blood glucose levels and mitigates postprandial hyperglycemia [79]. Inulin also stimulates the release of GLP-1, which enhances the secretion of insulin from the β-cells of the pancreas [80]. Furthermore, the reported secondary metabolites, such as coumarins and carotenoids, evince antioxidant properties which enhance the protection of pancreatic β-cells from oxidative stress caused by reactive oxygen species (ROS) and preserve the insulin-producing function of the pancreatic β-cells [58].

A previous study was conducted on the plant tubers to investigate the effect of the plant on glucose tolerance and the hepatic lipid profile. This study demonstrated that the intake of *H. tuberosus* L. tubers improved lipid metabolism and glucose tolerance in rats fed a high-fat diet (HFD). The tubers increased the excretion of triglycerides and total cholesterol into feces while reducing their levels in the liver and inhibiting fat and glycog en accumulation; the study proved that *H. tuberosus* L. can improve glucose tolerance and the hepatic lipid profile, thereby mitigating non-alcoholic fatty liver disease (NAFLD), which is closely related to type 2 diabetes mellitus (T2DM) [26]. According to Takahashi et al. [77], the oral administration of the plant helps in reducing postprandial hyperglycemia. This report is supported by Najima et al. [81], who investigated the efficacy of supplementing *H. tuberosus* L. to reduce postprandial plasma glucose levels in a randomized placebo-controlled study. The study results showed that a 12-week supplementation with *H. tuberosus* L., rich in inulin, significantly inhibited postprandial blood glucose increases compared to a placebo. Significant differences were observed between the groups at week 0 and week 12 in fasting plasma glucose (FPG) and 0.5 h OGTT (oral glucose tolerance test) values, while no differences were seen at 2 h OGTT. Overall, the supplement effectively mitigated early postprandial glucose increases, indicating its potential for improving glucose regulation. Another study investigated the antidiabetic potential of the ethyl acetate fraction of *H. tuberosus* L. using insulin-resistant HepG2 cells, a model for studying hepatic glucose uptake and insulin resistance. Insulin resistance is linked to impaired glucose uptake, contributing to hyperglycemia. Treatment with the fraction significantly improved glucose uptake in a concentration-dependent manner, with a 50 µg/mL dose increasing uptake by nearly 75% [37].

A protection mechanism study of the plant against T2DM, focusing on abnormal hepatic lipid metabolism in a high-fat diet, was conducted. A streptozotocin-induced diabetic mouse model was used to carry out this study. This study examined the protective effects of *H. tuberosus* L. inulin against type 2 diabetes mellitus (T2DM) associated with abnormal hepatic lipid metabolism and gut microbiota dysfunction in high-fat diet and streptozotocin-induced diabetic mice. Inulin supplementation significantly improved key biochemical markers of T2DM, including reductions in blood glucose, HbA1c, triglycerides, total cholesterol, low-density lipoprotein cholesterol, and serum pro-inflammatory cytokines. It also ameliorated abnormal hepatic lipid metabolism by modulating the expression of genes involved in lipid and cholesterol production and breakdown [82].

Furthermore, a study on *H. tuberosus* L. flowers revealed significant α-glucosidase inhibitory activity, linked to their phenolic compounds. The methanol extract and isolated compounds from *H. tuberosus* L. flowers were analyzed, with quercetin-3-O-β-D-glucopyranoside exhibiting the highest α-glucosidase inhibition (60.0% ± 10.3% at 250 μg/mL). T According to the author, this activity, along with antioxidant properties, was attributed to the presence of chlorogenic acid derivatives, flavonoids, and other phenols. These findings suggest that *H. tuberosus* L. flowers, particularly their phenolic ingredients, may have therapeutic potential for managing blood glucose levels [68]. Lastly, antidiabetic-related proteins, such as trehalose phosphorylase, thaumatin, profilin, and glyceraldehyde-3-phosphate, have been extracted from the plant [13].

*Helianthus tuberosus* L. may exert antidiabetic potential through mechanisms that improve insulin sensitivity and the regulation of insulin secretion and glucose metabolism, but the specific pathways are yet to be confirmed [37].

### 6.3. Antimicrobial Properties

*Helianthus tuberosus* L. is known to exert antimicrobial activities due to the presence of bioactive compounds and the fiber content. The antimicrobial compounds from the plant exhibit suppression of the growth of micro-organisms, including bacteria, viruses, and fungi [83]. The compounds act by disrupting the cell membranes of the micro-organisms, by interfering with their metabolic processes as well as by inhibiting their ability to form biofilms [84]. The dietary supplementation of *H. tuberosus* L. extract in growing Japanese quail significantly influenced intestinal microbiota in a study by Abdel-Wahab et al., promoting the growth of beneficial bacteria like *Lactobacilli* and Bifidobacteria while reducing harmful bacteria such as *E. coli* and *Salmonella*. The data showed a marked increase in *Lactobacilli* populations and a decrease in pathogenic bacteria with the supplementation of *H. tuberosus* L., particularly at 200 and 400 ppm concentrations. These results suggest that *H. tuberosus* L. can improve gut health by promoting beneficial microbiota and inhibiting harmful pathogens, thereby supporting overall health and immunity in quail [63].

A study examined the effectiveness of a symbiotic composition, combining *Bifidobacterium bifidum* with fructans from *Helianthus tuberosus* L., against *Staphylococcus aureus*. The results showed that *B. bifidum* combined with *H. tuberosus* L., precipitated with 20% ethanol, demonstrated the most significant growth inhibition of *S. aureus*. The study also noted that the efficacy of the probiotic–prebiotic combination was dependent on the initial bacterial count ratio and fermentation duration. These results suggest that the prebiotic activity of *H. tuberosus* L. could be used to effectively control pathogenic bacteria like *S. aureus* in certain conditions [85]. The antimicrobial properties of *H. tuberosus* L. were investigated in the context of wound healing. The study tested the ethyl acetate fraction for antibacterial activity against common wound-associated pathogens, such as *Staphylococcus aureus*, *Pseudomonas aeruginosa*, *Bacillus subtilis*, and *Klebsiella pneumoniae*. The ethyl acetate fraction exhibited a strong antibacterial activity, with the largest zone of inhibition observed against *Staphylococcus aureus* (17.35 ± 1.34 µg/mL). The inhibition activities against other pathogens were *Klebsiella pneumoniae* (16.34 ± 1.09 µg/mL), *Bacillus subtilis* (12.48 ± 1.27 µg/mL), and *Pseudomonas aeruginosa* (8.26 ± 0.61 µg/mL) [37]. The results confirmed significant antimicrobial activity. The antimicrobial activity of previously isolated compounds of *H. tuberosus* L. was evaluated against various pathogens. Compounds 1 and 14 (ent-kaur-16-en-19-oic acid and β-sitostenone) exhibited antimicrobial effects against *Enterococcus faecium*, with MIC values ranging from 6.25 to 12.50 μg/mL. β-sitostenone also showed anti-TB activity with an MIC of 25.00 μg/mL against *Mycobacterium tuberculosis*. These results highlight the potential antimicrobial properties of these compounds, especially in combating bacterial infections and tuberculosis [61].

The antifungal potential of the *H. tuberosus* L. extracts was demonstrated against *Rhizoctoniasolani, Alternarisolani*, and *Botrytis cinerea* [73]. The antifungal and antibacterial activities of *H. tuberosus* L. were further demonstrated by the plant extract obtained using a combination of water and carbon dioxide against bacterial (*Staphylococcus aureus, Escherichia coli*) and fungal (*Candida albicans*, *Candida glabrata*) strains. The CO_2_ + H_2_O extract showed notable antimicrobial activity, with MIC values ranging from 0.62 to 5 mg/mL against bacteria and 5 to 10 mg/mL against yeasts. The highest activity was observed against *Staphylococcus aureus* ATCC 29213, with an MIC of 2.5 mg/mL.

Furthermore, the antiviral properties of *Helianthus tuberosus* L. were demonstrated as inhibitory properties using the respiratory syncytial virus (which usually affects the human respiratory system of infants and the elderly) replication in mouse models. The study first evaluated the anti-respiratory syncytial virus activity of Jerusalem artichoke polysaccharide (JAP), an inulin-type polysaccharide extracted from *H. tuberosus* L. In vitro, JAP exhibited an inhibitory effect on RSV that was similar to that of the standard antiviral drug ribavirin, with an IC_50_ value of 29.15 ± 0.44 μg/mL and a selectivity index (SI) of 68.27, compared to ribavirin’s IC_50_ of 30.19 ± 0.23 μg/mL and SI of 76.21. In vivo, JAP significantly improved weight loss and reduced lung injury in RSV-infected mice in a dose-dependent manner. It also inhibited RSV replication, as evidenced by a reduction in RSV mRNA levels in lung tissue, highlighting its potential as an antiviral agent against RSV [67]. Lastly, the plant has abundant phenolic compounds, including quercetin 7-O-glucoside and kaempferol 3-O-glucoside, which are known to collectively display a broad spectrum of antimicrobial potential against fungi, bacteria, and some viruses [37].

### 6.4. Antioxidant Properties

*Helianthus tuberosus* L. exhibits its antioxidant activities predominantly through the bioactive compounds found in the plant. The abundance of phenolic compounds and flavonoids, such as coumarins, caffeic acid, and caffeoylquinic acid, confers higher antioxidant activity [66]. These phenols are helpful in scavenging free radicals and reactive oxygen species (ROS). They neutralize free radicals generated in cells by accepting and donating electrons, which is essential in reducing oxidative stress and cellular damage in the body [86]. This subsequently reduces the risk of several cardiovascular and metabolic diseases, including cancer, diabetes, obesity, heart disease, stroke, cognitive decline, and atherosclerosis [66].

The antioxidant activities of two flavonoids from *H. tuberosus* L. leaves were evaluated through DPPH, ABTS^+^, and hydroxyl free radical scavenging assays at concentrations ranging from 0.5 to 200.0 µg/mL. The results showed that both flavonoids exhibited significant scavenging activity, with SC_50_ values (the concentration required to scavenge 50% of free radicals) lower than the positive control, butylated hydroxytoluene (BHT), in all the assays. The first flavonoid had SC_50_ values of 90.61 µg/mL (DPPH), 1.40 µg/mL (ABTS), and 15.07 µg/mL (OH), while the second flavonoid had values of 106.80 µg/mL (DPPH), 1.31 µg/mL (ABTS), and 10.61 µg/mL (OH). These findings indicate that the flavonoids from *H. tuberosus* L. leaves have strong antioxidant capacities, outperforming the synthetic antioxidant BHT in certain assays [31]. The results proved that the leaves of *H. tuberosus* L. exhibit stronger antioxidant effects compared to the positive control BHT and could be used to protect the cells therapeutically from oxidative damage. *Helianthus tuberosus* L. reportedly possesses different types of antioxidant enzymes (catalase, peroxidase, superoxide dismutase, and glutathione dehydrogenase) [87]. These enzymes are known to neutralize various types of reactive oxygen species [88]. Another study was conducted to determine the total phenolic and total flavonoid contents in the plant extracts (leaves and tubers). Folin–Ciocalteau reagent and aluminum nitrate non-anhydrite were used, respectively, to measure the quantity of these antioxidants. The study measured the total phenolic content (TPC) and total flavonoid content (TFC) of ethanolic extracts from tubers and leaves using calibration curves of gallic acid and quercetin, respectively. The results showed that the leaf extracts had significantly higher phenolic and flavonoid content than the tuber extracts, with the TPC and TFC in the leaves being 5.07 and 7.14 times higher, respectively. Additionally, both extracts demonstrated a dose-dependent increase in TPC and TFC [69].

Furthermore, Showkat et al. [65] compared the radical scavenging activity of phenolic acids from various parts of *Helianthus tuberosus* L. The results showed that the ethanol extracts of the leaves exhibited the strongest radical scavenging activity (454, 374, and 321 µg/mL). This was followed by the flower (229, 238, and 169 µg/mL) and tuber extracts (155, 141 and 112 µg/mL), while the stem extracts (64, 70, and 38 µg/mL) showed the lowest inhibition capacity. These results demonstrate the antioxidant potential of the plant.

The plant also exerts its antioxidant properties through vitamins. Vitamins C and E, are present in *H. tuberosus* L., and account for the antioxidant activity of the plant [89]. Overall, the antioxidant properties exhibited by this plant help in improving cardiovascular, metabolic, and gut health [90].

### 6.5. Anti-Inflammatory Properties

Inflammation mainly happens when the human body responds to the infection, caused primarily by stress, pathogens, or tissue damage. Prolonged infection might lead to serious health disorders, such as cancers, obesity, arthritis, and diabetes mellitus; hence, it is imperative to study the anti-inflammatory potentials of various compounds [91,92]. *Helianthus tuberosus* L. exhibits anti-inflammatory properties which enable it to prevent inflammation in the body. The plant possesses abundant phenolic compounds, such as caffeic acid, caffeoylquinic acid, and quercetin-7-O-glucoside, which exert anti-inflammatory properties [37,63,93]. The high content of inulin also help in growing beneficial bacteria of the gut, which enhance the immune response and subsequently reduce inflammation associated with immune deficiency [94].

Onoja et al. [95] evaluated the anti-inflammatory potential of *H. tuberosus* L. in rat models. A leaf methanol extract was used on the egg albumin-induced paw oedema model. The plant extract exhibited a dose-dependent anti-inflammatory response. A treatment of 300 mg/kg of the extract caused a 33.33% decrease in inflammation, while the control (acetylsalicylic acid) caused a 36.36% decrease in inflammation. Another study investigated the anti-inflammatory effect of the *H. tuberosus* L. tuber extract using the A549 human lung epithelial cells. To measure activity, nitric oxide production was evaluated on the cells. The ethanol extract exhibited a strong inhibitory effect on nitric oxide production, which is a key mediator of inflammation. This inhibition was dose-dependent, as the extract concentration increased. These findings highlight the potent anti-inflammatory activity of *H. tuberosus* L. ethanol extract, making it a promising candidate for managing inflammatory conditions [96].

Furthermore, methyl 2-(4-methoxy-4-oxobutanamide) benzoate, an isolated compound from *H. tuberosus* L., effectively mitigates macrophage inflammation and its downstream effects. At concentrations up to 100 μg/mL, it showed no cytotoxicity in RAW 264.7 cells and significantly reduced the expression of inflammatory markers, including MCP-1, Rantes, IL-1β, IL-6, and TNF-α, at both the transcriptional and protein levels. Furthermore, it suppressed the activation of the MAPKs and NF-κB pathways, key regulators of inflammation and insulin resistance. These results highlight the potential to attenuate inflammation by targeting macrophage-driven inflammatory responses by methyl 2-(4-methoxy-4-oxobutanamide) benzoate [70]. Lastly, Gupta and Chaturvedi [73] reported that inulin from the plant also inhibits overexpression of G-protein coupled receptor 43, which manages high-fat diet-induced obesity by lowering the level of circulating liposaccharides and lowers C-reactive protein levels to attenuate inflammation.

### 6.6. Anticancer Properties

Alternately, researchers have explored the therapeutic potential of medicinal plants for the treatment of cancer as they are known to display fewer side effects compared to the modern anticancer medications [97]. *Helianthus tuberosus* L. is one of these medicinal plants that have shown potential in exerting anticancer activity [98]. *Helianthus tuberosus* L. exerts anticancer activities that are mainly due to the presence of compounds in the plant that can inhibit the growth of cancer cells, including chlorogenic acid, dicaffeoylquinic acid, isoxazolidine, and caffeic acid [37,59,66]. According to Wang et al. [28], toxic chemical carcinogens in the body can also be removed by compounds found in the plant aerial parts. Compounds displaying antioxidant and anti-inflammatory activities also prevent the formation of tumors and the growth of cancer cells [99]. The compounds also neutralize free radicals that can cause DNA damage [87,96]. The plant’s ability to exert anticancer activities is attributed to the rich abundance of these compounds.

A study was carried out by Beyazyüz et al. [100] to evaluate the anticancer properties of *Helianthus tuberosus* L. using tuber and shell extracts on MCF-7 breast cancer cells. Both extracts demonstrated significant cytotoxic effects in a dose-dependent manner while showing no toxicity on normal MCF-12A breast epithelial cells. The tuber extract exhibited stronger effects in reducing cell proliferation, disrupting mitochondrial membrane potential and inducing early and late apoptosis, particularly after 72 h of application. Antimetastatic analyses revealed that the tuber extract effectively reduced cancer cell adhesion and invasion rates, with the shell extract also showing notable activity. The study identified inulin and bioactive compounds like sesquiterpene lactones and flavonoids as contributors to these effects. Overall, the results suggest that *H. tuberosus* L. extracts possess antimetastatic and apoptosis-inducing properties, making them promising candidates for breast cancer therapy. According to Afoakwah et al. [78], *H. tuberosus* L. tuber extracts demonstrated significant anticancer activity by inhibiting the proliferation of HT-29 colon cancer cells in a dose-dependent manner, with suppression reaching 78.05 ± 3.9% at a dose of 250 μg/mL. The Annexin V assay revealed that the tuber extracts induced DNA fragmentation and late apoptosis in the HT-29 cell line. Additionally, the extracts caused cell cycle arrest at the G1 phase, ultimately leading to programmed cell death. These findings highlight the potential of tuber extracts as a natural therapeutic agent for colon cancer. It has also been reported that *H. tuberosus* L. has strong inhibitory effects on human tumor cells, such as DLDI colon cancer cells, SKOV3 ovarian cancer cells, and Caco2 colon cancer cells [28].

### 6.7. Neuroprotective Effects

The plant exerts its neuroprotective effects primarily through its antioxidant and anti-inflammatory properties [101]. The *Helianthus tuberosus* L. plant consists of a variety of classes of bioactive compounds that are essential to combat oxidative stress and neuroinflammation, which are key factors in neurodegenerative diseases, such as Alzheimer’s disease [58]. *Helianthus tuberosus* L. comprises proteins that are important in managing Alzheimer’s disease, Parkinson’s disease, and Huntington’s disease [39].

The plant can inhibit the aggregation of amyloid-beta plaques (a hallmark of Alzheimer’s disease) by inhibiting the activity of the β-secretase enzyme. An ethanol plant extract of *H. tuberosus* L. also inhibited the activity of the cholinesterase enzymes, which subsequently improved cholinergic transmission in the brain. Moreover, a study on the glycogen synthase kinase- 3β enzyme, an enzyme involved in the generation of tau tangles, which is another hallmark of Alzheimer’s disease was carried out, and the plant inhibited its activity [102]. These results show that *H. tuberosus* L. demonstrates significant potential as a neuroprotective agent through its antioxidant and anti-inflammatory properties, as well as its ability to target key enzymes and pathways implicated in neurodegenerative diseases such as Alzheimer’s disease.

### 6.8. Wound Healing Properties

Wound healing is the process in which the body repairs damaged tissues to ensure normal structure and function of the tissues. Infections, chronic diseases, poor blood circulation, and age can cause slow healing in humans [103]. The primary influences that slow down the wound healing process are the presence of aerobic bacterial species and the production of an excessive amount of reactive oxygen species that prolong the inflammatory stage [104]. *Helianthus tuberosus* L. has been traditionally used by folk medicine practitioners [28].

The wound healing potential of *H. tuberosus* L. is primarily exhibited by the essential components, including inulin, flavonoids, and phenolic compounds. These compounds display antimicrobial, anti-inflammatory, and tissue-regenerating effects [28]. The compounds reduce swelling and pain at the wound sites, fight harmful bacteria that can cause infections on the wounds, and, lastly, stimulate tissue regeneration on the damaged tissues. An in vitro wound healing scratch assay was conducted using the fibroblast NIH3T3 cells to determine the wound healing potential of *H. tuberosus* L. The cells were treated with different concentrations of *H. tuberosus* L. with an incubation period of 48 h. The results demonstrated the wound healing potential of the plant. An *H. tuberosus* L. ethyl acetate fraction demonstrated significant wound healing potential in a fibroblast-based scratch assay using NIH 3T3 cells. The extract was tested at concentrations of 25, 50, and 100 µg/mL, and it promoted cell migration in a time-dependent manner over 48 h. Compared to untreated control cells, the treated cells showed enhanced wound closure, with the highest activity observed at 100 µg/mL. These findings suggest that *H. tuberosus* L. can accelerate the wound healing process by stimulating fibroblast migration, making it a promising candidate for treating chronic wounds [37].

**Figure 3:**
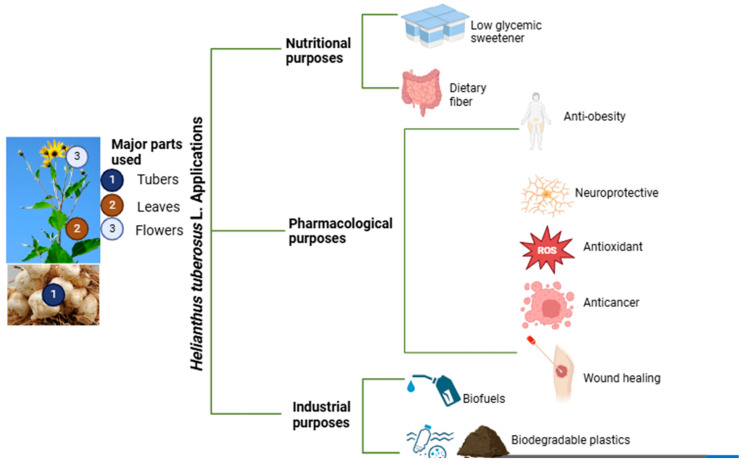
Schematic representation of the applications of *Helianthus tuberosus* L. parts.

Furthermore, another study evaluated the wound healing properties of *H. tuberosus* L. powder, prepared via freeze-drying. In the experiment on mice, the topical application of *H. tuberosus* L. powder significantly improved wound contraction rates and enhanced the healing process, as confirmed by histopathological analysis. The treated group showed notably better outcomes compared to the untreated control group, demonstrating the potential of *H. tuberosus* L. powder as an effective agent for wound healing [105]. Figure 3 outlines the summary of all the applications of *H. tuberosus* L. that are discussed in the paper.

## 7. Toxicity Assessment of *Helianthus tuberosus* L.

Preparations of the plant around the globe as food and in folk medicine have shown no adverse side effects [106]. A study conducted cytotoxicity assessments using the methanol extract of the plant tuber on the XTT assay. The MCF-12A breast epithelial cell line was used, and the cells exhibited no sign of toxicity [100].

Similarly, the chemical contaminant composition was determined in the plant tubers. The results indicated that the plant has low levels of contaminants; this confirms that its consumption may not lead to any health problems that may be caused by contaminants [53]. The plant is however only safe when taken in small amount; excessive consumption may lead to abdominal discomfort, bloating, and flatulence for individuals with low tolerance to inulin and fructose [107]; so, necessary precautions are advisable when consuming it.

## 8. Gaps in *Helianthus tuberosus* L. Research

There is still a lack of research on *H. tuberosus* L., including research on drug interactions with other prescribed medications, as well as comprehensive studies on its potential side effects and the adverse reactions that may occur from using it as a medicinal component. Previous studies have mainly focused on its potential therapeutic properties without fully analyzing the long-term negative properties that may rise from consuming the plant [30,108].

There is limited information on the drug–drug interactions involving *Helianthus tuberosus* L. Conducting more studies on the drug interactions with other medications might be the first step to help prevent any adverse reactions or any interference with existing treatment plans. The information on scientific studies on clinical trials is insufficient. Evaluation of the effectiveness of *Helianthus tuberosus* L. in treating and managing specific health conditions in human studies is needed to support its use in healthcare. Clinical trials will help in determining the safety and effectiveness of the plant in people affected by several diseases.

More research is needed to identify all the bioactive compounds that are responsible for their pharmacological properties and their mechanism of action. More research on the mechanisms through which the bioactive compounds in *H. tuberosus* L. act will deepen understanding of its potential benefits and applications. Moreover, there is a need to establish dosing guidelines to achieve consistent and effective results. Lastly, there is limited understanding regarding the synergistic effects of the various identified compounds present in *H. tuberosus* L. and how they can be used together to produce therapeutic outcomes.

Further research into this gap could enhance our understanding of the plant’s full potential and safety for human health.

## 9. Future Prospectives

The future of *Helianthus tuberosus* L. lies in its potential for commercialization, social impact, and scientific innovation. Its applications range from its use as a functional food ingredient to its use in the pharmaceutical and biofuel industries [21,24,35], leveraging its high inulin content and bioactive compounds, like phenolic acids and flavonoids [67]. These applications cater to the rising demand for prebiotics and natural therapeutics, while its potential as a bioethanol [23] source positions it as a renewable energy solution. The cultivation of *H. tuberosus* L. can significantly improve the economic and social status of rural communities by creating income opportunities and promoting sustainable agriculture. Moreover, its diverse pharmacological properties, including antioxidant, anti-inflammatory, and anticancer effects, make it a strong candidate for drug discovery against chronic diseases like diabetes and cancer [59,60,61,62]. Environmentally, the plant’s low-input nature, soil-enriching capabilities, and adaptability to harsh conditions [8,24] strengthen its attractiveness. Investment in research, genetic improvement, and policy support is critical to maximizing its benefits and ensure its integration into sustainable development strategies.

## 10. Conclusions

The present review summarizes the traditional use, medicinal properties, phytochemistry, nutritional value, toxicity assessments, and science gaps of *Helianthus tuberosus* L. (Jerusalem artichoke). The Jerusalem artichoke is a rich source of inulin, which is an indigestible polysaccharide that acts as a functional food and also plays an important role in exhibiting pharmacological properties. Inulin exerts anti-obesity, antidiabetic, and antimicrobial properties, among others [33].

Previous trials have shown through analytical procedures, such as HPLC, GC-MS/MS, HPLC-UV, HPLC-NMR, and LC-MS/MS, that *Helianthus tuberosus* L. has a variety of bioactive compounds (e.g., phenolic acids, coumarins, flavonoids, and sesquiterpenes), which are responsible for the different pharmacological properties outlined in the review paper [28,60,61,62]. *Helianthus tuberosus* L. is easily accessible in various parts of the world and has been reported to be non-toxic [100]. It therefore represents a promising source for the discovery of new and non-/less toxic bioactive natural compounds to be used as hits or leads for drug development. However, more research on *Helianthus tuberosus* L. is needed to close the research gaps associated with the plant’s therapeutic potential as well as to validate the medicinal attribute of the plant in modern medicine.

## Figures and Tables

**Table 1:** Synonyms and common names of *Helianthus tuberosus* L.

Synonyms	Vernacular Names	
	Country/Region	Name
*Helianthus tomentos* [11,12]	Japan	Kikuimo [13]
*Helianthus serotinus* [14]	USA	Sunroot [11]
*Helianthus esculentus* [15]	Europe	Fusichoke [11]
*Helianthus tuberosus var subcamesces* [16]	Italy	Girasole [17]
	USA	Sunchoke [11]
	Germany	Earthapple [11]
	Canada	Canada potato [11]
	France	Topinambur [18]
	USA	Jerusalem artichoke [19]

**Table 2:** Nutritional composition of *Helianthus tuberosus* L. tubers.

Nutrient	Quantity (per 100 g Fresh Weight)	Method of Quantification
Water	77–80%	Oven drying method [30,51]
Dry matter	16.5–36.2%	Drying to constant weight [9,30]
Protein	2–3%	Benstein method [30,52]
Nitrogen	1.850 mg	Kjedahl method [9,30]
Total fat	0.73–0.76 g	Soxhlet extraction [30]
Dietary fiber	2.4–20.8 g	Enzymatic–gravimetric method [30,53]
Inulin	14–25%	Solvent extraction, hydrolysis, and spectrophotometry [30,54]
Vitamin C	6.0 mg	Titration with 2.6 dichlorophenolindophenol sodium salt [29,30]
Calcium (Ca)	89 mg	Inductively coupled plasma spectrometry (ICP-AES) [30,51,52]
Iron (Fe)	5.10 mg	ICP-AES [30,51]
Magnesium (Mg)	26.0 mg	ICP-AES [30,51]
Phosphorus (P)	117 mg	ICP-AES [30,51,52]
Potassium (K)	644 mg	ICP-AES [30,51,52]
Sodium (Na)	6.00 mg	ICP-AES [30,51]

**Table 3:** Phytochemicals reported in different parts of *Helianthus tuberosus* L.

Plant Part	Phytochemical	Chemical Structure	Chemical Class	Pharmacological Properties
Leaf	Kaempferol 3-O-glucoside	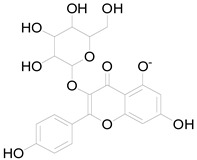	Flavonoid	Antioxidant, anti-inflammatory, antimicrobial [58,63].
	Quercertin-7-O-glucoside	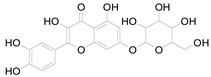	Flavonoid	Antioxidant, anti-inflammatory, antimicrobial [63,64].
	Butein	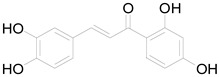	Flavonoid	Anticancer, anti-inflammatory [58].
	Pendunculin	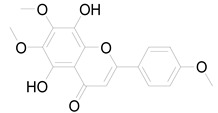	Flavonoid	Antioxidant, anti-inflammatory [31].
	Budlein A	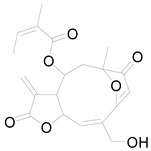	Sesquiterpenoid	Anti-inflammatory, antioxidant [28].
	Isotripliciolide tiglate	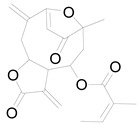	Sesquiterpenoid	Anticancer, anti-inflammatory, antioxidant [62].
	Caffeic acid	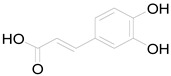	Phenolic acid	Anti-inflammatory, antioxidant, antitumor [37].
	Feruyloquinic acid	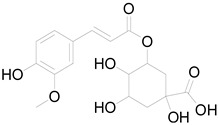	Phenolic acid	Antioxidant, antimicrobial, antiviral, antidiabetic [37,59].
	6-methoxy-7-hydroxy-coumarin	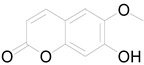	Coumarin	Antibacterial, antifungal, antiparasitic, anti-inflammatory [58,64].
Tuber	Caffeoylquinic acid	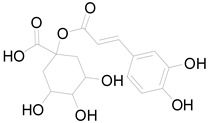	Phenolic acid	Anti-inflammatory, antioxidant [37].
	Ferulic acid	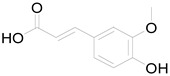	Phenolic acid	Antioxidant, anti-inflammatory, anticancer, antidiabetic [65].
	Chlorogenic acid	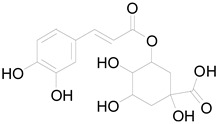	Phenolic acid	Antioxidant, anti-inflammatory, antibacterial [59,66].
	Ayapin	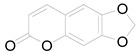	Coumarin	Antioxidant, anti-inflammatory, antimutagenic [58,64].
	6-methoxy-7-hydroxy-coumarin	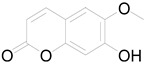	Coumarin	Antibacterial, antifungal, antiparasitic, anti-inflammatory [58,64].
Flower	Kaurenoic acid	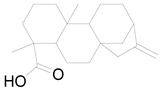	Terpenoid	Antibacterial, anti-inflammatory, antioxidant, antimicrobial [61].
	Faradiol	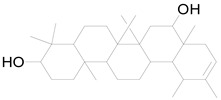	Terpenoid	Antimicrobial, antioxidant, anti-inflammatory, antidiabetic, antihyperlipidemic [67].
	Loliolide	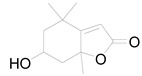	Terpenoid	Antimicrobial, antitumor, anti-inflammatory [64].
	8-Methoxyobliquin	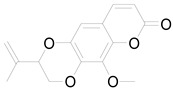	Coumarin	Anticancer, antiviral, antioxidant [67].
	7-O-Prenylscopoletin	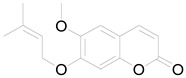	Coumarin	Neuroprotective effects [67].
	Sulferein glycoside	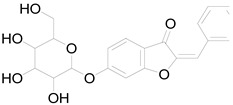	Flavonoid	Antiviral, antioxidant, antidiabetic [67].
Whole plant	Vanillin	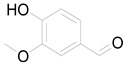	Flavonoid	Anticancer, antidiabetic, antimicrobial, anti-inflammatory [58].
	Puerarin	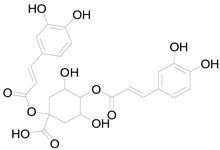	Flavonoid	Antioxidant, anti-inflammatory [28].
	Quercetin-3-0-β-D-glucopyraside	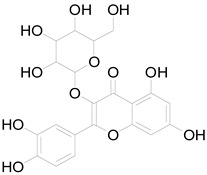	Flavonoid	Antimicrobial, anti-inflammatory, antioxidant [68].
	Hymenoxin	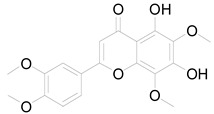	Flavonoid	Antimicrobial [28].
	Rhamazin	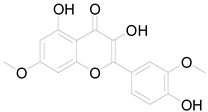	Flavonoid	Anticancer, antioxidant, anti-inflammatory [28].
	Nevadensin	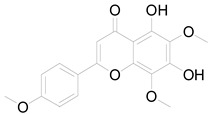	Flavonoid	Anti-inflammatory, antihypertensive, antibacterial [28].
	1,4-dicaffeoylquinic acid	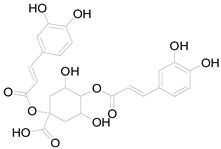	Phenolic acid	Antiviral, antioxidant, anti-inflammatory [37].
	P-coumaroylquinic acid	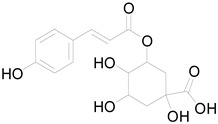	Phenolic acid	Antibacterial, antioxidant, anti-inflammatory [28].
	Salicylic acid	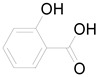	Phenolic acid	Wound healing properties, anti-inflammatory, antioxidant [28].
	4,15-isoatriplicolide	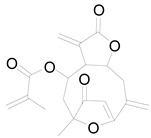	Sesquiterpene	Anticancer [69].
	18-dihydrobudlein A	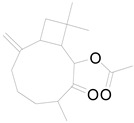	Sesquiterpene	Antimicrobial [28].
	Heliannuol A	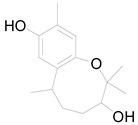	Sesquiterpene	Antimicrobial [28].
	Heliangin	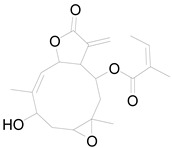	Sesquiterpene	Anticancer, anti-inflammatory, antimicrobial [70].
	Eupatolide	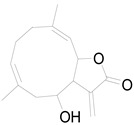	Sesquiterpene	Anticancer [28].
	Niveusin B	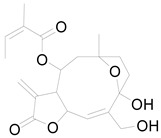	Sesquiterpene lactone	Antimicrobial [28].
	Methyl 2-(4′-methoxy-4′-oxobutanamide) benzoate	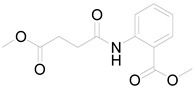		Anti-inflammatory, antioxidant [70].
